# Anxiety and Leisure-Domain Physical Activity Frequency, Duration, and Intensity During Covid-19 Pandemic

**DOI:** 10.3389/fpsyg.2020.603770

**Published:** 2020-12-29

**Authors:** Cassio M. Meira, Kaique S. Meneguelli, Maysa P. G. Leopoldo, Alex A. Florindo

**Affiliations:** School of Arts, Sciences, and Humanities, University of São Paulo, São Paulo, Brazil

**Keywords:** exercise, sport, anxious, personality, coronavirus, individuality

## Abstract

This study investigated relationships between state anxiety and leisure-domain physical activity levels during Covid-19 pandemic. We used frequency, duration, and intensity as key variables of physical activity. Trait anxiety, state anxiety before pandemic, age, gender, and education level were also included in the analysis. Our general hypothesis was that participants who declared doing more physical activity levels would exhibit lower levels of anxiety during the Covid-19 pandemic. A convenient sample of 571 volunteer adults (mean age 39 ± 14 years) was drawn mainly from São Paulo State (89.2% of the sample), the epicenter of Covid-19 pandemic in Brazil. To obtain the participants’ levels of anxiety (trait, state before pandemic, and state during pandemic) we used a validated short-version of the *State and Trait Anxiety Inventory*. Levels of physical activity were measured via questions from VIGITEL, a validated questionnaire about the individual’s habits on risk factors. Answers were given regarding the first week of March 2020 (before pandemic) and at the very moment the participant was filling in the electronic form (June 2020). Data analyses were conducted through descriptive and inferential techniques, with the use of non-parametric tests and linear regression models. Overall, participants’ responses indicate that anxiety levels were higher during the pandemic compared to the period that preceded the pandemic, and that frequent and long physical activity in the leisure-domain reduced anxiety, regardless its intensity. The regression models revealed an inverse relationship between physical activity and anxiety (the more physical activity, the less anxiety) and independent of gender, age, education level, trait anxiety, and physical activity before pandemic.

## Introduction

High levels of anxiety have been regarded as a factor that provokes feelings of personal vulnerability because it negatively affects well-being and everyday functioning (Asmundson et al., 2010). In addition, increased anxiety might threaten the individual’s health by increasing risk perception (Köteles and Simor, 2014) and leading to depression (World Health Organization, 2019). Anxiety is a negative emotion to situations perceived as dangerous or stressful characterized by subjective feelings of nervousness, worry, and unease that is related to the activation of the autonomic system, e.g., heart rate, perspiration, breathing (Spielberger and Reheiser, 2009). Taken as a state, a regulatory factor of anxiety is control perception, the degree to which individuals believe having resources and capacity to face an imposed challenge (Cheng et al., 2009). For example, individuals with high levels of anxiety tend to respond to perceived threatening situations with reactions that are disproportionate to the objective danger when compared to those with low levels (Spielberger and Reheiser, 2009).

Although high anxiety is often associated with negative performance on cognitive tasks (Eysenck et al., 2007), it has not always provoked negative effects as indicated by Jones (1995), who makes a distinction between debilitating and facilitating dimensions of the anxiety response. Joneś model of anxiety is centered on the degree of control the individual is able to exert over the self and the environment. Those who have self-perception to be capable of being in control and to achieving the goals tend to fathom anxiety symptoms as facilitative, whereas the ones who regard themselves as unable to control themselves and thus have diminished expectations about goal achievement are prone to construe as debilitative the anxiety effects. According to Jones’ model, the individual might interpret the stressor in the environment as either a challenge or a threat, influencing differentially physical activity and anxiety levels. The perception of the stressor according to individual differences leads to either a positive or a negative perception of control under which the individual is optimistic or pessimist, respectively. If optimistic, individuals are able to deal with the stressor and to reach their goals. If pessimistic, they are not capable of handling the stressor and reaching the goals. Sport studies have showed that the athletes who interpret stressors as a challenge are more inclined to exhibit superior performance (Weinberg and Gould, 2017).

The Covid-19 pandemic is a current global health problem that has been plaguing the world population. To minimize the health damages of the disease, most countries’ governments adopted measures of social distancing and home isolation. These measures are thought to decrease contagion rates of coronavirus (Bedford et al., 2020), at the expense of impacts on mental health of individuals, for instance raising the incidence of symptoms of anxiety and depression (Brooks et al., 2020). Fear is another mental health symptom that has been associated to Covid-19 (Ornell et al., 2020). These restraining factors are potential factors that give rise to the possibility of being contaminated and being constrained at home. This unknown and detained environment condition characterizes uncertainty, which is regarded as a stressor for individuals (Weinberg and Gould, 2017). The stress response to the risk of contamination and confinement situation might give rise to distinct levels of anxiety and lead to various misbehaviors (Muniyappa and Gubbi, 2020; Pfefferbaum and North, 2020; Qiu et al., 2020; Rajkumar, 2020; Torales et al., 2020), including the ones related to physical activity (McGrath, 1970). Physical activity is thought to be a positive mediator of anxiety and, in some cases, even an individual who exhibits high anxiety can sustain high levels of physical activity, for instance, by activating compensatory mechanisms (Eysenck et al., 2007). We adopted leisure-domain physical activity definition from the 2020 WHO guidelines on physical activity (Bull et al., 2020): *physical activity performed by an individual that is not required as an essential activity of daily living and is performed at the discretion of the individual.*

It is widely known that physical active is an effective “remedy” for reducing anxiety levels. Physiological studies have developed some hypotheses to explain the beneficial effects of physical activity on anxiety: it produces protective effects on neurogenesis (Moylan et al., 2013), potentially reduces hyperactivity of the sympathetic nervous system affecting abnormalities in the fear conditioning processing (Asmundson et al., 2013), and tends to activate anti-inflammatory mechanisms to diminish oxidative stress (Viana and Andrade, 2012). Research syntheses (Jayakody et al., 2014; Gordon et al., 2017; Stubbs et al., 2017) and cross-sectional studies (Vancini et al., 2017) have indicated physical activity as a positive mediator for anxiety in patients and practitioners. During the Covid-19 pandemic, behavioral studies have advocated for physical activity as a positive aspect when coping with anxiety. Creese et al. (2020) compared prior (from 2015 to 2019) longitudinal mental health data of individuals with data collected from the same individuals during the Covid-19 pandemic. The findings demonstrated that loneliness and diminished physical activity levels were associated to a poor mental health during the pandemic. These studies indicate that physical activity might be closely linked to mental health, especially anxiety. As the Covid-19 pandemic is likely to increase anxiety, it is important to thoroughly scrutinize levels of physical activity and relate them to levels of anxiety during the health problem we are facing in 2020. As far as we know, the association of anxiety with frequency and duration of physical activity during the Covid-19 pandemic was not tackled by any study.

Thus, the purpose of the present study was to examine relationships between state anxiety and leisure-domain physical activity levels during Covid-19 pandemic. We used frequency, duration, and intensity as key variables of physical activity. Trait anxiety, state anxiety before pandemic, age, gender, and education level were also included in the analysis. We set a general hypothesis: participants who report performing more physical activity exhibit lower levels of anxiety during the Covid-19 pandemic. The imposing restrictions that the pandemic encompasses could alter the way individuals perceive control (Jones, 1995), with effects on their levels of anxiety and physical activity. If interpreted as a challenge, individuals feel able to control the possibility to be infected by coronavirus so that we hypothesize that they will keep or increase physical activity levels. Conversely, as long as individuals are not capable of assuming effective control over being contaminated by coronavirus, we believe they will reduce the levels of physical activity. Given that personal and environmental mediating factors may have significant effects on the evaluation by individuals about stress factors (Jones, 1995), we also examined relevant variables in the pandemic scenario, such as age, gender, and education level.

## Materials and Methods

### Participants

This was a cross-sectional study with a convenient sample of 571 adult volunteers, 200 male and 371 female, mean age of 39 ± 14 years, was selected mainly from São Paulo State (89.2% of the participants), the epicenter of Covid-19 pandemic in Brazil. The state of São Paulo (Brazil), whose estimated population in 2020 is 46 million people, had 200, half million, and 1 million Covid-19 cases in 18th March, 15th June, and 16th August, respectively. By 21st November 2020, there have been in the state more than 1.2 million cases and more than 41.000 deaths caused by Covid-19^1^.

The presence of comorbidity(ies) was stated by 21.7% of the sample (no participant was excluded due to this criterion) and 69.9% had completed tertiary education. We excluded 19 participants who had not yet completed 18 years of age and one participant who did not fill out the anxiety and physical activity questions.

All participants read and signed an electronic consent form prior taking part in the study, which was approved by the University’s Ethics Committee under the protocol CAAE 33639720.0.0000.5390.

### Instruments and Procedure

Due to time restraints reasons, to measure anxiety we used a validated short version of the *State and Trait Anxiety Inventory-Y* (Spielberger et al., 1983), a traditional self-report instrument extensively used in research and clinical settings across different cultures. The Brazilian short-version is the *STAI-S-6*, whose psychometric properties were reported as appropriate for the six questions of each anxiety scale – State Anxiety: Cronbach α coefficient was 0.75 and full-length and short-form STAI-S-6 version correlation was 0.90; Trait Anxiety: Cronbach α coefficient was 0.73 and full-length and short-form STAI-S-6 version correlation was 0.89 (Fioravanti-Bastos et al., 2011). To respond to the state scale participants are required to describe how they feel at “this very moment” about the following items: I am worried; I am tense; I feel nervous; I feel calm; I am relaxed; I feel at ease. Participants describe how they “generally” feel about the trait scale “I feel secure; I worry too much over something that really doesn’t matter; I feel nervous and restless; I get in a state of tension or turmoil as I think over my recent concerns and interests; I am calm, cool and collected; I make decisions easily.” The answers are given along a four-point Likert intensity scale: 1 = not at all, 2 = somewhat, 3 = moderately, 4 = very much. The score of each answer adds up to the final score of anxiety.

In order to obtain leisure-domain physical activity data, we used the 2019 version of the Surveillance System of Risk and Protective Factors for Chronic Diseases by Telephone Survey (VIGITEL), an official (Ministry of Health – Brazil) self-report instrument based on non-face-to-face interviews available online at https://portalarquivos.saude.gov.br/images/pdf/2020/April/27/vigitel-brasil-2019-vigilancia-fatores-risco.pdf. VIGITEL was validated by Monteiro et al. (2008). Its leisure-domain physical activity section has five questions about the practice of physical exercise or sport in the last 3 months (yes/no), the main type of activity (one choice among multiple options), whether the practice is done once at least once a week (yes/no), the weekly frequency of the activity (one choice among multiple options), and the daily duration of the practice (one choice among multiple options). Participants’ responses were organized according to the following variables (yes/no): physical activity practice, 40 min or more, three or more times per week, three or more times per week during at least 40 min. The intensity of physical activity was obtained according to the type(s) of activity(ies) self-declared by the participant; we labeled the categories “moderate” and “vigorous or vigorous + moderate” as specified by the compendium of physical activities (Ainsworth et al., 2011). Moreira et al. (2017) reported reliability (k) of 0.7 for active individuals in leisure time and 0.64 for inactive individuals and regarding validity, there were no significant differences for leisure-domain physical activity in the VIGITEL frequencies (32.8%) when compared to those of the World Health Organization (2015)’s Global Physical Activity Questionnaire – GPAQ (30.8%), *p* = 0.538. We chose VIGITEL because it holds simple and short questions with objective and direct answers and involves no cost.

In the first week of June 2020 we invited in private approximately 800 individuals (response rate of 71.4%) to answer the questionnaires via social media (Facebook, Instagram, and Whatsapp) and email. The outbreak of the pandemic in the State of São Paulo, when social restrictions came into effect, began in the third week of March 2020. To give answers about behaviors before the pandemic, the participants were instructed to recall behaviors from the first week of March 2020. The responses about the period during the pandemic were given at the very moment the participants were filling in the electronic form, that is, during June 2020. We collected the answers via electronic devices through a Google electronic form designed exactly as the original questionnaires. The missing data were reported at the end of the tables.

### Data Analysis

Data were organized in Microsoft Excel sheets. Analyzes were threefold. Firstly, we ran a descriptive analysis that reported absolute and relative (percentage) frequencies of participants on each variable of interest. Then, due to non-normal distributions measured by Kolmogorov–Smirnov tests, to compare different levels of physical activity frequency, duration, and intensity on levels of state anxiety during Covid-19 pandemic we used non-parametric techniques (separate Kruskal–Wallis and Mann–Whitney tests). The Wilcoxon test was employed to compare anxiety levels before and during pandemic. Lastly, four linear regression models were conducted to establish the influence of leisure-domain physical activity during the pandemic in state anxiety during the pandemic. Although homoscedasticity Durbin–Watson tests pointed out violations of this assumption for some models, we ran the analyzes with the original data because regression analyzes are robust for any distribution of data in large samples (Lumley et al., 2002). We initially ran unadjusted models and then adjusted models by gender, age range, education level, and anxiety range. Analyzes were conducted in IBM SPSS Statistics 24.0 version and Stata version 16.1 StataCorp LCC. The alpha level was set at 5% for all analyzes.

## Results

### Descriptive and Inferential Analysis

Descriptive values of absolute and relative frequencies are shown in Table 1. The sample is particularly adult, female, highly educated, and from the State of São Paulo, Brazil.

**TABLE 1:** Frequencies of social demographics, anxiety, and physical activity variables.

Variable	Categories	*n*	%
Gender	Male	200	35
	Female	371	65
Age (range)	18–29	172	30.1
	30–39	112	19.6
	40–49	159	27.8
	50–76	128	22.5
Education level	Incomplete tertiary −	172	30.1
	Complete tertiary +	399	69.9
**Physical activity practice before pandemic**			
Once a week*	Yes	475	94.4
	No	27	5.6
3 times or more/week^∞^	Yes	367	75.5
	No	119	24.5
40 min or more/day^#^	Yes	424	86.9
	No	64	13.1
3 times or more/week and 40 min or more/day^×^	Yes	328	65.3
	No	174	34.7
**Physical activity practice during pandemic**			
Once a week**	Yes	382	86.1
	No	58	13.9
3 times or more/week∧	Yes	267	67.9
	No	126	32.1
40 min or more/day^*circ*^	Yes	259	65.6
	No	136	34.4
3 times or more/week and 40 min or more/day^∼^	Yes	185	47.2
	No	207	52.8

Missing responses: *69, ^∞^85, ^#^83, ^×^69, **131, ∧78, °176, ^∼^179.

With reference to anxiety data, the Wilcoxon test for paired samples (before x during pandemic) indicated a significant effect (*Z* = −9.51; *p* < 0.0001), with higher values during the Covid-19 pandemic. We also ran comparisons of state anxiety levels during Covid-19 pandemic. The Kruskal–Wallis analysis showed a significant effect for age [χ^2^ (3) = 44.41; *p* < 0.0001], with higher values for the younger age ranges. The Mann-Whitney analysis indicated a significant effect for gender, with women exhibiting higher anxiety compared to men (*U* = 29,410; *p* < 0.0001). The test detected significant effect for education level, with the ones who completed tertiary education self-reporting lower anxiety compared to those who did not finished higher education (*U* = 27,001.5; *p* < 0.0001). There were no differences between those who declared having comorbidity and the ones with no reported comorbidity (*U* = 25,767.5; *p* = 0.404). The descriptive and *p*-values for these variables are presented in Table 2.

**TABLE 2:** Descriptive statistics of anxiety and statistical differences of state anxiety during Covid-19 pandemic (total sample and stratified by age, gender, and education level).

	Trait anxiety (Mean ± SD)	State anxiety before pandemic (Mean ± SD)	State anxiety during pandemic (Mean ± SD)	*p*
Total sample	13.78 ± 3.66	13.30 ± 3.81	15.33 ± 4.16	<0.0001*
Age				<0.0001^#^
18–29	15.45 ± 3.92	13.64 ± 3.84	16.74 ± 3.9	
30–39	13.57 ± 3.22	13.53 ± 4.14	15.76 ± 4.1	
40–49	13 ± 3.17	12.87 ± 3.71	14.8 ± 4.11	
50–76	12.19 ± 3.14	13.18 ± 3.58	13.73 ± 3.98	
Gender				<0.0001^#^
Male	12.8 ± 3.36	12.5 ± 3.5	14.35 ± 3.93	
Female	14.13 ± 3.69	13.73 ± 3.9	15.87 ± 4.2	
Education level				<0.0001^#^
Incomplete tertiary −	15.13 ± 3.94	13.91 ± 3.68	16.39 ± 4.21	
Complete tertiary +	13.04 ± 3.3	13.04 ± 3.84	14.88 ± 4.1	

*p-values of the comparison state anxiety before pandemic x state anxiety during pandemic; ^#^*p*-values of comparisons during pandemic.

Regarding leisure-domain physical activity during Covid-19 pandemic (Table 3), Mann-Whitney tests revealed no differences in the level of anxiety between those who practiced *versus* the ones who did not practice physical activity (*U* = 9919; *p* = 0.198). However, the tests pointed out significant low levels of anxiety for participants who practiced physical activity for (1) 40 min or more per session compared to those who practiced less than 40 min per session (*U* = 14607.5; *p* = 0.005), (2) three or more days per week compared to the ones who practiced less than 3 days per week (*U* = 12760.5; *p* < 0.0001), and (3) at least 40 min per session on three or more days of the week in comparison to those who practiced less than this and also those who did not practice at all (*U* = 14616.5; *p* < 0.0001). No differences were detected between those who engaged in moderate physical activity compared to the ones who took vigorous or vigorous and moderate physical activity (*U* = 18714; *p* = 0.119).

**TABLE 3:** Descriptive statistics and statistical differences of anxiety according to leisure-domain physical activity during Covid-19 pandemic.

	Trait anxiety (Mean ± SD)	State anxiety before pandemic (Mean ± SD)	State anxiety during pandemic (Mean ± SD)	*p*
Practice once a week				=0.198
Yes	13.59 ± 3.59	13.37 ± 3.87	15.11 ± 4	
No	13.48 ± 3.67	12.69 ± 3.47	15.93 ± 4.54	
40 min per day				=0.005*
Yes	13.39 ± 3.63	13.30 ± 3.76	14.77 ± 4.03	
No	13.82 ± 3.52	13.38 ± 3.93	15.82 ± 3.9	
3 days/week				<0.001*
Yes	13.34 ± 3.63	13.37 ± 3.97	14.57 ± 3.94	
No	14.02 ± 3.49	13.25 ± 3.55	16.33 ± 3.88	
40 min/day on 3 days or +				<0.001*
Yes	13.64 ± 3.52	13.23 ± 3.83	15.87 ± 4.09	
No	13.44 ± 3.73	13.33 ± 3.8	14.3 ± 3.9	
Intensity				=0.119
Moderate	13.62 ± 3.70	13.11 ± 3.93	15.48 ± 4.19	
Vigorous/vigorous + moderate	13.61 ± 3.52	13.48 ± 3.73	14.85 ± 3.85	

*Significant effect: *p* < 0.05 (all comparisons were performed only among the participants who practiced physical activity during the Covid-19 pandemic).

### Linear Regression Models

We carried out four linear regression models (Table 4) to establish the influence of leisure-domain physical activity (practice, frequency, duration, frequency, and intensity of physical activity as predictors) on state anxiety during the pandemic (dependent variable) controlled by physical activity and state anxiety before pandemic, trait anxiety, gender, age, and education level. Homoscedasticity was checked through the test of Durbin–Watson in all models. Violations of this assumption were detected for the unadjusted models that combined duration and frequency of physical activity and for all adjusted models. As regression analyzes are valid for any distribution of data in large samples (Lumley et al., 2002), we ran the analyzes with the original data.

**TABLE 4:** Linear regression results examining the association between state anxiety and leisure-domain physical activity among participants during Covid-19 pandemic.

			State anxiety scores			
	Leisure-domain physical					
Main effect	activity	Model	β scores	CI 95% (scores)	*p*-value	R^2^
*F* (1,438) = 2.06	Practice (once a week)	Unadjusted	−0.82	−1.95; 0.3	0.152	0.005
*F* (11,402) = 17.65		Adjusted	−0.93	−2; 0.14	0.087	0.33
*F* (1,393) = 6.12	Duration (at least 40 min per session)	Unadjusted	−1.04	−1.87; −0.21	0.014	0.02
*F* (11,364) = 15.68		Adjusted*	−1.04	−1.81; −0.27	0.008	0.32
*F* (1,391) = 17.22	Frequency (at least 3 times per week)	Unadjusted	−1.76	−2.59; −0.93	0.0001	0.04
*F* (11,362) = 16.64		Adjusted∧	−1.57	−2.32; −0.81	0.0001	0.34
*F* (1,440) = 16.34	Duration and Frequency (at least 3 times/week for 40 min/day)	Unadjusted	−1.57	−2.33; −0.81	0.0001	0.04
*F* (11,404) = 20.11		Adjusted^#^	−1.78	−2.49; −1.07	0.0001	0.35
*F* (1,405) = 2.43	Intensity (moderate/vigorous)	Unadjusted	−0.63	−1.42; 0.16	0.12	0.006
*F* (11,375) = 15.99		Adjusted	−0.7	−1.43; 0.03	0.06	0.32

Main contributors: *gender, state anxiety before pandemic and trait anxiety; ∧ state anxiety before pandemic and trait anxiety; ^#^physical activity practice, gender, state anxiety before pandemic and trait anxiety.

The first and the last regression models were not significant for physical activity practice (once a week) during pandemic and for intensity of physical activity as predictors of state anxiety during Covid-19 pandemic. The remaining regression models indicate that state anxiety during Covid-19 pandemic can be predicted by duration (40 min or more per day), frequency (three or more days per week) and the combination of physical activity duration and frequency (40 min or more a day, three times a week). The analysis demonstrated an inverse relationship between physical activity and anxiety (the more physical activity, the less anxiety), independent of gender, age, education level, trait anxiety, and physical activity before pandemic.

## Discussion

Our aim in the present study was to examine relationships between state anxiety and leisure-domain physical activity levels during Covid-19 pandemic. We used frequency, duration, and intensity as key variables of physical activity and conveniently selected a Brazilian sample, mainly from the State São Paulo, because this region was the epicenter of the pandemic in the country. Overall, our findings were in line with our general expectation that individuals who declare performing more physical activity show lower levels of anxiety. Practice of physical activity *per se* showed no significant statistical association, but there was a significant effect in the comparison of the group that practiced physical activity for 40 min or more with the group that practiced less than 40 min. The results were stronger for the group that practiced physical activity for at least 40 min on 3 days or more of the week as compared to the group that practiced less than this or those who did not practice physical activity. Even more impressive were the results of the group that practiced physical activity for at least 40 min on five or more days of the week as compared to the group that practiced less than three times a week for 40 min. Surprisingly, those who practiced moderate physical activities did not differ in terms of anxiety levels from the ones who practiced vigorous or vigorous/vigorous + moderate physical activities. These findings appear to give evidence that not “any” or “general” physical activity was capable of lessening anxiety. Thus, frequency and duration of physical activity seems to play a pivotal role in reducing anxiety levels during Covid-19 pandemic.

As identified by the participants’ self-reports, specifications for physical activity regarding frequency and duration were crucial to reduce anxiety during Covid-19 pandemic. In fact, other studies and institutions advocate frequent and lengthy physical activity. World Health Organization (Bull et al., 2020) recommended at least 150 min per week of moderate to vigorous physical activity, and during the Covid-19 pandemic, WHO recommended that people maintain their physical activity levels. In addition, the study carried out by Moore et al. (2012) with many dataset of cohort showed a dose-response between leisure-domain physical activity and mortality. The participants who do not perform frequent and lengthy physical activity during the pandemic tend to exhibit higher levels of anxiety, thus provoking negative feelings that affect well-being and everyday functioning (Asmundson et al., 2010) as well as increasing risk perception (Köteles and Simor, 2014). These higher levels of anxiety may lead to personal vulnerability and depression (World Health Organization, 2019; Muniyappa and Gubbi, 2020; Pfefferbaum and North, 2020; Qiu et al., 2020; Rajkumar, 2020; Torales et al., 2020). Absent or little exercise might degrade even more the perception of the pandemic danger by the individual, who may not feel capable of facing the challenges imposed by the pandemic (Cheng et al., 2009), even producing disproportionate reactions, e.g., elevated heart rate, sweat, and breathing (Spielberger and Reheiser, 2009). Built upon the degree of control the individual is able to exert over the self and the environment, Jones (1995) model might be a credible explanation for the findings of the current study. His model differentiates anxiety into a debilitating and a facilitating dimension. Self-perceptions to be in control and to achieve the goals tend to fathom anxiety symptoms as facilitative, and this might be the case for the participants who practice more physical activity as compared to the ones who practice less or no physical activity, these latter probably regard themselves as having little control during the pandemic, hence exhibiting debilitative anxiety effects.

Research syntheses have provided support for physical activity as a key component in dealing with anxiety. When compared with regular treatments (antidepressants and/or psychological techniques), physical activity, regardless of type and intensity, had an important role in diminishing patients’ anxiety symptoms, though not as effective as the administration of antidepressants (Jayakody et al., 2014). Further, Stubbs et al. (2017) pointed out that aerobic and moderate-to-high intense exercise decreased anxiety symptoms more than control conditions in individuals who have currently been diagnosed with anxiety and stress-related disorders. Moreover, Gordon et al. (2017) revealed that resistance exercise training reduced anxiety symptoms, especially on healthy participants compared to the ones with physical or mental illnesses and that effect sizes did not vary according to duration, frequency, and intensity of exercise or gender, age, and strength improvement. Thus, physical activity seems to be a contributing anxiolytic to treat anxious patients and appear to be a crucial factor to allay anxiety of healthy individuals.

In addition, cross-sectional studies give considerable evidence that physical activity can reduce anxiety levels. Vancini et al. (2017) addressed in obese and overweight participants the effects of an aerobic physical activity program (walking) versus a mixed program (Pilates method) on levels of depression, trait and state anxiety, and quality of life. The programs were held for 8 weeks, 3 days a week, with session durations of 60 min. Both programs exerted marked effects on quality of life, depression, and trait-anxiety, but the aerobic program surprisingly increased state-anxiety. These latter findings are not in line with prior research on the positive role of aerobic physical activity in anxiety (Gordon et al., 2017). In addition, Creese et al. (2020) investigated the relationship between anxiety and physical activity during the Covid-19 pandemic and demonstrated isolation and low levels of physical activity were related to poor mental states when compared to the pre-pandemic period. Taken together, these findings tend to support physical activity as a distinctive feature to overcome anxiety.

Our cross-sectional study has some limitations that should be pointed out. The participants of our convenience sample filled out the questionnaires on their own via the electronic form so that they had to recollect behaviors, what might pose a recall bias problem. As in other anxiety scales, STAI only measures the intensity of symptoms that represent the presence of anxiety, not the interpretation of symptoms as positive or negative about behaviors or performance. We did not measure the perception of control related to stressors in the environment; therefore, even though we feel it is useful to explain the findings, the reasoning employed to discuss this concept should be taken as speculative. Additionally, the education level of more than 2/3 of the sample is high (complete tertiary), thereby, it is noteworthy to highlight that highly educated people tend to show high levels of physical activity as compared with the general population. In spite of these limitations, we believe the present study makes a valuable contribution to the field with the indication that regular (at least three times a week for 40 min a day) leisure-domain physical activity can be an ally against anxiety during the Covid-19 pandemic.

## Conclusion

Self-declared reports on leisure-domain physical activity and anxiety give evidence that during the Covid-19 pandemic that the practice *per se* (once a week) of physical activity is not sufficient to reduce anxiety levels. It is important that individuals take frequent and lengthy physical activity to diminish levels of anxiety. In particular, moderate and/or vigorous physical activity performed regularly at least 3 days a week for 40 min per session seems to exert anxiolytic effects during Covid-19 pandemic.

## Data Availability Statement

The raw data supporting the conclusions of this article will be made available by the authors, without undue reservation.

## Ethics Statement

The studies involving human participants were reviewed and approved by the Ethics Committee for Research with Human Beings, School of Arts, Sciences and Humanities, University of São Paulo. Written informed consent to participate in this study was provided by the participants’.

## Author Contributions

CM: conception, data organization, and writing. KM: data collection, data organization, and writing. ML: data organization, formatting, and writing. AF: data analysis and writing. All authors contributed to the article and approved the submitted version.

## Conflict of Interest

The authors declare that the research was conducted in the absence of any commercial or financial relationships that could be construed as a potential conflict of interest. The handling editor declared a shared affiliation, though no other collaboration, with the authors.

## Footnotes

1 https://www.seade.gov.br/coronavirus
